# A Reproducible Protocol and Framework for Large Language Model (LLM)-Assisted Estrogen and Progesterone Receptor (ER/PR) Scoring

**DOI:** 10.7759/cureus.108708

**Published:** 2026-05-12

**Authors:** Svetoslav Bardarov, Alireza Zarineh

**Affiliations:** 1 Pathology and Laboratory Medicine, Richmond University Medical Center, New York City, USA; 2 Pathology, Rutgers Robert Wood Johnson Medical School, Newark, USA

**Keywords:** ai, breast cancer, digital pathology, hormone receptors, large language models

## Abstract

Estrogen and progesterone receptor (ER/PR) scoring in breast cancer is vulnerable to interobserver variability, particularly at diagnostic thresholds. While large language models (LLMs) offer potential as ancillary tools, no standardized deployment framework exists. This is a feasibility and protocol-development study that establishes a reproducible protocol for LLM implementation in pathology labs. Using College of American Pathologists (CAP) proficiency-testing tissue microarrays, we developed a systematic framework that combines recursive prompt engineering and zero-state reset (ZSR) protocols, requiring fresh chat sessions for each evaluation to prevent conversational bias. Three models were evaluated: Claude Haiku 4.5 (Anthropic, San Francisco, CA, USA), Gemini 3.0 (Google, Mountain View, CA, USA), and Gemma 3.0 12B (Google, Mountain View, CA, USA). The ZSR protocol proved essential, improving accuracy from a 70-80% baseline to ≥95% CAP concordance. Performance varied across models and scoring approaches: Claude achieved the highest concordance (90-98%), followed by Gemini (85-100%) and Gemma (73-93%), demonstrating that systematic prompt refinement, not model capacity, drives diagnostic accuracy. Final concordance exceeded 83% across all models. The locally hosted model approached cloud-level performance within a clinically meaningful range, achieving concordance rates that, while numerically lower, remained within acceptable feasibility thresholds for a protocol development context. We identified systematic error sources, including image artifacts, slide contamination, and compression effects. This protocol provides pathology laboratories with evidence-based guidance for implementing LLM-assisted ER/PR scoring without requiring specialized infrastructure or extensive computational resources. The framework is immediately actionable as a research and quality-assurance tool and provides a reproducible foundation for future clinical validation studies across laboratory settings.

## Introduction

Breast carcinoma is a significant global health issue. It is the most commonly diagnosed cancer and a leading cause of cancer-related deaths among women worldwide. In the United States, projections for 2026 estimate over 320,000 new cases of invasive breast cancer and around 42,000 deaths. Despite a decrease in mortality rates by over 40% since the late 20th century due to improvements in early detection and treatment, the rising number of cases, particularly among younger women, highlights the ongoing need for accurate diagnostic and prognostic assessment [1,2].

A critical part of breast cancer management is the immunohistochemical (IHC) assessment of hormone receptors, namely the estrogen receptor (ER) and progesterone receptor (PR). There is broad agreement in the medical community that ER and PR status are among the strongest prognostic factors and are essential for treatment decisions [3]. The presence of these receptors indicates that patients are likely to benefit from hormone therapies such as selective ER modulators or aromatase inhibitors. On the other hand, the lack of these markers often requires more intensive chemotherapy treatments [4]. To ensure consistent interpretation of the brown 3,3'-diaminobenzidine (DAB) chromogen signal in IHC, pathologists use established semi-quantitative scoring systems. The Allred Score is a popular method that combines a proportion score (0-5), reflecting the percentage of positive cells, with an intensity score (0-3) for a total score range of 0 to 8 [5]. Alternatively, the H-Score (or Histoscore) provides a more detailed evaluation by multiplying the percentage of cells at each intensity level (0, 1+, 2+, 3+) by its intensity value, yielding a continuous scale from 0 to 300 [6]. These systems aim to turn complex visual color information into reproducible clinical categories. Even with these standardized methods, visual inspection of ER and PR can still reveal significant differences between observers. Telling apart “negative” from "weak" (1+) staining intensity is subjective, especially when humans use approximation to determine the actual percentage of positive cells. Human perception of DAB intensity can be influenced by factors such as counterstain contrast, tissue thickness, edge-effect artifacts, and individual variability in color perception. This variability is especially noticeable at the "low-positive" threshold (1-10%), where small differences in visual assessment can result in different classifications. Such discrepancies could affect a patient's chances of receiving vital hormone therapy [7,8].

Computational pathology might offer a solution to these reproducibility problems. While traditional convolutional neural networks have already succeeded in specific image analysis tasks, the rise of large language models (LLMs) with multimodal capabilities, especially smaller, locally run models, opens up new possibilities for diagnostic support. In this study, we propose that both commercial and locally hosted LLMs can help improve the consistency of ER and PR evaluations. We analyzed performance both qualitatively, by assigning 0, 1+, 2+, or 3+ intensity, and semi-qualitatively using a three-tier system: negative, low expression, and high expression. By comparing these AI-assisted interpretations with expert pathologists' consensus, we want to see whether these tools can provide a reliable, standardized ancillary study for the pathology process. Rather than merely testing LLMs' ability to score ER/PR staining, we aimed to establish a feasible protocol and a standardized framework for their deployment across pathology laboratories to ensure reproducible results.

## Materials and methods

College of American Pathologists (CAP) proficiency testing tissue microarray (TMA)

The study utilizes TMA sets provided by the CAP as part of the ER/PR survey program. The evaluation consisted of four TMA slides (two for ER and two for PR), each containing 10 cores of invasive carcinoma, for a total of 20 cores per analyte. The 10 cases used for iterative prompt optimization were drawn from the larger 80-case pool and were included in the final concordance analysis. No independent held-out validation cohort was employed; this is a recognized limitation explicitly acknowledged in the Discussion. Future studies should incorporate a fully separated test set to permit unbiased performance estimation. CAP consensus reference values were derived from the proficiency testing summary reports accompanying each TMA core set. Consensus was defined as the modal scoring category assigned by the majority of participating laboratories across the ~1,500 CAP-enrolled sites, each evaluating 20 cores per challenge set.

For ER, the most frequently utilized primary antibody was the rabbit monoclonal clone SP1 (used by ~70% of participants), followed by 6F11 and EP1. For PR, the dominant clone was 1E2, with others including 16, 636, and 1294.

Image acquisition

Tissue microarray images were acquired at 200× magnification with an Olympus BX46 microscope (Olympus Corporation, Tokyo, Japan) and an Olympus DP70 digital camera (Olympus Corporation, Tokyo, Japan). The camera utilized a 2/3-inch CCD sensor with piezo-shifted imaging technology, providing approximately 1.45 megapixels of effective resolution and producing up to 12.5 million-pixel-equivalent still images at 4080 × 3072 pixels with color interpolation. Image capture was performed at 12-bit per channel (R/G/B) color depth (48-bit total) with ISO equivalent sensitivity set to 200. Auto white balance was enabled, and fixed exposure settings were maintained throughout all acquisitions to ensure standardization across samples. All images were saved as JPEG format with 80% compression and stored for subsequent analysis. All slides were thoroughly wiped with alcohol pads and lens paper to ensure they were free of fingerprints and dust particles.

Hardware and software configuration for local LLM evaluation

For local evaluation of Gemma 3.0 12B (gemma-3-12b-it-Q4-K-M.GGUF, 12.0 GB; Google, Mountain View, CA, USA), a Beelink GTi14 AI mini PC (Shenzhen AZW Technology Co., Ltd., Guangdong, China) with an Intel Core Ultra 9 185H (16T/22C, up to 5.1GHz; Intel Corporation, Santa Clara, CA, USA), 64GB DDR5, a 2TB PCIe4.0 SSD, and a mini desktop computer were used. GPU acceleration was provided by an ASUS TUF Gaming GeForce RTX 5070 graphics card (12 GB GDDR7, PCIe 5.0; ASUS, Taipei, Taiwan). Gemma was executed using LM Studio version 0.4.2 (build 2), achieving inference speeds of 40-50 tokens per second. This configuration enabled rapid local model inference without reliance on cloud-based APIs.

Cloud-based LLMs

Claude Haiku 4.5 (Anthropic, San Francisco, CA, USA) and Gemini 3.0 (Google, Mountain View, CA, USA) were accessed via their respective free-tier web interfaces (claude.ai and gemini.google.com). Testing was conducted between February and April 2026. At the time of access, the available model versions were Claude and Gemini. Images were uploaded directly via the browser-based file upload function without manual resizing; however, platform-side compression behavior cannot be fully ruled out, posing a reproducibility caveat. Each case was submitted in a single independent session with no carry-over context (zero-state reset (ZSR) protocol). No repeated runs per case were performed beyond the iterative prompt refinement cycles described above. Interface settings were default; no system-level configuration options were available through the free-tier interface.

Statistical analysis

Concordance rates are reported as simple percentage agreement with the CAP consensus. Formal statistical measures, such as Cohen's kappa or confidence intervals, were not calculated given the feasibility study design and sample size. Future validation studies should incorporate kappa statistics, sensitivity/specificity per scoring tier, and inter-rater reliability metrics.

## Results

Case selection and sample preparation

A total of 40 ER and 40 PR cases were selected from TMA CAP proficiency testing samples with established consensus classifications. All 80 images were captured at 200× magnification and saved as JPEGs with 80% compression, as the evaluated LLM platforms currently support this format. In contrast, the uncompressed TIFF format is not yet compatible with LLM image analysis capabilities.

Recursive prompt engineering (RPE) and LLM evaluation protocol

Each LLM (Claude, Gemini, and Gemma) was evaluated using two distinct scoring approaches, qualitative intensity grading and semi-qualitative tiered classification, each subjected to independent iterative prompt refinement cycles. For both approaches, a batch of 10 control cases selected from the 80 clinical cases served as internal validation controls throughout the optimization process. Model predictions were compared with the established CAP proficiency-testing consensus, and cases with discordant classifications were identified. Detailed feedback was provided to the model, specifying which cases were incorrectly interpreted and explaining the nature of each misclassification (e.g., failure to distinguish between scoring categories or misinterpretation of staining intensity or distribution). Based on this error analysis, each model was explicitly asked to refine and improve the original prompt to address the identified discrepancies. The refined prompt was then applied to re-evaluate the same 10 control cases in a new, separate chat session to avoid internal language model learning bias and context accumulation. This iterative cycle of evaluation, error feedback, prompt refinement, and re-evaluation in isolated chat sessions continued until either a minimum of 95% concordance with CAP consensus was achieved or no further improvement was observed across consecutive rounds.

Qualitative prompt refinement (0, 1+, 2+, 3+ intensity scoring)

In the qualitative approach, models were tasked with assigning a discrete intensity score (0, 1+, 2+, or 3+) to each IHC image based on DAB chromogen depth relative to the hematoxylin counterstain. Prompt iterations focused on calibrating the perceptual boundary between intensity grades, with particular emphasis on distinguishing faint 1+ staining from negative (0) cases at the critical 1% threshold. The final refined prompts for each model after convergence are summarized in Table 1.

**Table 1:** Iterative LLM-derived qualitative prompts LLM: large language model, IHC: immunohistochemistry, DAB: 3,3′-diaminobenzidine

Qualitative prompt		
Gemini (round 3)	Claude (round 5)	Gemma (round 3)
Role: expert breast pathologist (IHC specialist). Task: quantitatively and qualitatively evaluate estrogen receptor (ER) and progesterone receptor (PR) expression in digital images of tumor tissue. Scoring logic and intensity calibration: evaluate the DAB (brown) chromogen against the hematoxylin (blue) counterstain using the following strict hierarchy: 3+ (high expression): deep, dark "chocolate" brown. The staining must be opaque, meaning it is dense enough to obscure the underlying nuclear chromatin details. 2+ (moderate expression): clear, distinct brown staining. Easily visible at low magnification, but retains a degree of translucency where the nuclear texture or blue counterstain is still subtly perceptible. 1+ (low expression): faint, light brown, or "ghost-like" nuclear staining. Critical rule: if the intensity is pale/faint, it must be classified as 1+ regardless of the percentage of cells stained 0 (negative): complete absence of nuclear brown staining. Only the blue counterstain is visible. Required reporting format: for every image analyzed, provide the following: intensity score: (0, 1+, 2+, 3+), proportion: estimated percentage (%) of positive tumor nuclei. Note: if in doubt between 2+ and 3+, opt for the aggressive site and call it 3+.	Role: you are an expert pathologist evaluating estrogen receptor (ER) and progesterone receptor (PR) expressions in breast tumors using IHC with DAB chromogen. You will score images using a 0, 1+, 2+, 3+ system based on visual inspection. Scoring criteria: 0 (negative): no nuclear staining or <1% positive tumor cells. Only blue (hematoxylin) counterstain visible in nuclei. 1+ (weak): very faint, barely perceptible brown nuclear staining. Light tan/pale brown that's difficult to appreciate. Minimal chromogen deposition, if in doubt, between 0 and 1+, use 1+. 2+ (moderate): use sparingly. Reserve for truly borderline cases only if uncertain between 1+ and 2+, use 2+ if uncertain between 2+ and 3+, use 3+. 3+ (strong): any clear, definite brown nuclear staining visible. Includes medium brown intensity. Do NOT require very dark/intense brown. If brown chromogen is clearly visible in tumor cell nuclei with more than 1% of positive cells, score as 3+. Clear brown = 3+, not just dark brown. Critical rules: focus on nuclear staining only (ER and PR are nuclear receptors).	Role: expert breast pathologist (IHC specialist). Task: quantitatively and qualitatively evaluate estrogen receptor (ER) expression in digital images of tumor tissue. Scoring logic and intensity calibration: evaluate the DAB (brown) chromogen against the hematoxylin (blue) counterstain using the following strict hierarchy: 3+ (high expression): deep, dark "chocolate" brown. The staining must be opaque, meaning it is dense enough to obscure the underlying nuclear chromatin details. 2+ (moderate expression): clear, distinct brown staining. Easily visible at low magnification, but retains a degree of translucency where the nuclear texture or blue counterstain is still subtly perceptible. 1+ (low expression): faint, light brown, or "ghost-like" nuclear staining. Critical rule: if the intensity is pale/faint, it must be classified as 1+ regardless of the percentage of cells stained 0 (negative): complete absence of nuclear brown staining. Only the blue counterstain is visible. Required reporting format: for each analyzed image, provide the following: intensity score (0, 1+, 2+, 3+). Proportion: estimated percentage (%) of positive tumor nuclei. Note 1: if in doubt between 2+ and 3+, opt for the more aggressive site and call it 3+. Note 2: if the cellularity is sparse but still more than 1%, analyze the depth of the brown intensity and opt for a higher score.

Semi-qualitative prompt refinement (negative/low/high expression)

In the semi-qualitative approach, models were tasked with classifying each case into one of three clinically meaningful tiers: negative, low expression, or high expression, based on the combined assessment of DAB positivity percentage and staining intensity. Prompt iterations in this paradigm focused on hierarchical decision logic, specifically on correctly tiering cases with faint 1+ staining versus cases with any moderate-to-strong (2+/3+) signal. The final refined prompts for each model after convergence are summarized in Table 2.

**Table 2:** Iterative LLM-derived semi-qualitative prompts LLM: large language model, IHC: immunohistochemistry, FFPE: formalin-fixed paraffin-embedded, DAB: 3,3′-diaminobenzidine

Semi-qualitative prompts		
Gemini (round 3)	Claude (round 5)	Gemma (round 3)
Role: you are an expert pathologist specializing in IHC scoring for estrogen receptor (ER) on FFPE tissue. Visual rule 1 (color): distinguish strictly between hematoxylin (blue) and DAB (brown). If a nucleus is only blue, it is NEGATIVE. Visual rule 2 (artifacts): Ignore "shadows" or dark blue clusters; only warm-toned brown counts as a signal. Scoring logic (aggressive high tiering): Negative (0): positive (brown) nuclei <1% or 0% total. Low expression: positive nuclei >1% AND intensity is strictly faint/transparent (1+). High expression: positive nuclei >1% AND intensity is opaque/dark (2+ or 3+). Note: any clear, solid brown signal that obscures the underlying blue is categorized as high.	Role: you are an expert pathologist specializing in IHC scoring for estrogen and progesterone receptors (ER and PR) on FFPE tissue. Visual rule 1 (color discrimination): hematoxylin counterstain = blue/purple = NEGATIVE. DAB chromogen = brown (warm-toned) = POSITIVE. If a nucleus is only blue, it is NEGATIVE. Visual rule 2 (artifact avoidance): ignore "shadows," dark blue clusters, or processing artifacts. Only warm-toned brown (tan, light brown, medium brown, dark brown) counts as a DAB signal. Visual rule 3 (enhanced 1+ detection - SENSITIVITY CHECKPOINT): 1+ staining: nuclei show a transparent, warm-brown tint. You can still see blue counterstain underneath, but there's a distinct tan/light brown overlay. Think: "brown-tinted blue" rather than pure blue. Key distinction: if the nucleus has ANY warm tone (tan, light brown, pinkish-brown) beyond pure cool blue/purple, count it as positive. Do NOT dismiss subtle DAB as an artifact. Faint brown is still brown. Intensity grading 0: no brown (pure blue/purple only). 1+: faint/transparent brown. Blue counterstain visible underneath, but warm overlay present. 2+: moderate opaque brown. Partially obscures blue counterstain. 3+: strong opaque brown. Completely obscures blue counterstain (dark chocolate brown). Scoring logic (aggressive high tiering). Negative (0): positive (brown) nuclei <1% OR 0%. Low expression: positive nuclei >1% AND intensity is all or predominantly 1+ (faint/transparent brown only). High expression: positive nuclei >1% AND intensity includes any areas with 2+ or 3+ (opaque brown). Note: any clear, solid brown signal that obscures the underlying blue qualifies as high, even if mixed with 1+ areas. Decision tree: count % positive nuclei (any warm brown tone). If <1% → NEGATIVE. If >1% → assess intensity: all/predominantly faint (1+) → LOW. Any moderate/strong (2-3+) present → HIGH.	ROLE: you are an expert pathologist evaluating estrogen receptor (ER) expression on FFPE tissues using IHC with DAB detection. Please follow these guidelines strictly: positive cell definition: a cell is considered ER positive if it exhibits visible brown nuclear staining attributable to the DAB reaction. Background staining or non-specific binding should be disregarded. Scoring thresholds (percentage of positive cells): negative (<1%): less than 1% of cells show detectable, specific brown nuclear staining or no brown stain at all. 1+ (low expression - 1-30%): between 1% and 30% of cells exhibit visible brown nuclear staining. The staining should be distinct from the background. 2+ (moderate expression - 31-69%): between 31% and 69% of cells show visible brown nuclear staining. 3+ (high expression - ≥70%): 70% or more of the cells exhibit visible brown nuclear staining. Ambiguity resolution: if you are uncertain whether a sample is negative or 1+, score it as 1+. If you are uncertain whether a sample is 1+ or 2+, score it as 2+. When assessing images with sparse cellularity, consider the potential for staining dilution and adjust your estimate accordingly. Prioritize assessment across multiple fields of view if available. Reporting: Report the ER expression as negative. Low expression: if the numerical score is 1+. High expression: if the numerical score is 2+ or 3+. The iterative refinement protocol converged by round 3, after which successive rounds showed no meaningful improvement, indicating the methodology had reached its effective ceiling with the tested prompts.

ER evaluation

The analysis of 40 ER cases demonstrates that prompt refinement is the critical determinant of diagnostic accuracy. Initially, all models exhibited a baseline "hesitancy" at the 1-10% threshold, often misclassifying low-positive cases as negative or vice versa due to a lack of explicit intensity-proportion weighting. However, after the third iteration of the RPE protocol, the models reached a plateau of near-perfect concordance with the CAP proficiency gold standard. Notably, the qualitative assessment of ER staining remained remarkably stable across all platforms; once the prompt defined the "warm brown tone" as the positive signal and established a rigid 1% cutoff, the LLMs effectively eliminated the visual subjectivity that typically plagues human interobserver reproducibility. This suggests that for ER, where the clinical stakes of a false negative are extremely high, LLMs can serve as a highly reliable ancillary study, provided they are prevented from "conversational drift" through fresh-state resets.

PR evaluation

PR scoring often presents a greater challenge in clinical practice due to more frequent heterogeneous staining patterns. Our findings from the 40 PR cases reflect this complexity while highlighting the robustness of the RPE approach. While initial rounds showed some discordance between the "high" and "low" semi-quantitative categories, the final prompt logic, which used a hierarchical decision tree to prioritize moderate-to-strong staining over faint staining, standardized the output across both cloud and local models. The performance of Gemma in the PR cohort is particularly noteworthy; despite its smaller parameter size, it achieved high concordance by strictly adhering to the prompt’s "rules of exclusion." These findings suggest that the model’s inherent “knowledge” is less important than the “logical framework” provided by the pathologist. Consequently, the high reproducibility observed in PR scoring indicates that LLMs can effectively circumvent traditional pitfalls in PR interpretation by applying consistent mathematical logic to visual data in pathology reports.

Mitigation of conversational bias (ZSR)

A critical finding of this study is the presence of "conversational inertia" or "contextual bias" in LLM architectures during batch image analysis. During the iterative refinement phase, it was observed that when multiple cases were processed within a single continuous chat thread, the models tended toward self-consistency rather than objective diagnostic accuracy. Specifically, the models’ attention mechanisms appeared to be "anchored" by preceding evaluations, leading to a degradation in performance on borderline cases (1% threshold) as the conversation length increased.

To mitigate this, we established a ZSR protocol that requires a fresh chat session for each evaluation round. This ensured that each case description was processed as an independent event, free from the logical "noise" or "drift" of previous scores. The implementation of ZSR was the turning point that enabled the models to move from an initial accuracy of 70-80% to expert-level concordance. This protocol is recommended as a standard requirement for any AI-assisted pathology auditing to ensure that the "rules" of the prompt are applied with absolute mathematical independence for every patient sample. Using this approach, all three models, regardless of parameter size or cloud dependency, achieved reproducible scoring results that were highly concordant with the established CAP consensus (Table 3).

**Table 3:** Qualitative and quantitative comparison between three LLM models after RPE LLM: large language model, RPE: recursive prompt engineering

Estrogen analysis			Progesterone analysis	
Model	Qualitative	Semi-qualitative	Qualitative	Semi-qualitative
Claude	90%	98%	88%	93%
Gemini	85%	88%	85%	100%
Gemma	75%	83%	73%	93%

## Discussion

Persistent discrepancies in biomarker interpretation have been documented at multiple levels, including inter‑observer differences in scoring thresholds, challenges at the low‑positive range, and variability in how borderline cases are categorized. A large nationwide population‑based study by Acs et al. demonstrated that variability in ER, PR, and HER2 assessment can lead to divergent therapeutic recommendations, particularly in low‑positive and equivocal categories, underscoring the clinical consequences of inconsistent scoring [9]. Even in the context of proficiency testing, pathologists frequently exhibit the greatest variability precisely in the 1-10% low‑positive ER/PR range, where subjective perception of DAB intensity and rough estimates of tumor cell percentage can lead to discordant classifications. These observations highlight a critical need for tools that standardize interpretation, especially at decision‑making thresholds where small scoring differences have disproportionate therapeutic impact [10].

Over the past decade, the rapid maturation of digital pathology and AI has provided a technical foundation for such standardization. Whole‑slide imaging is now sufficiently robust for primary diagnosis in many jurisdictions, and the parallel development of deep learning‑based image analysis has demonstrated that algorithmic scoring can approximate or exceed human performance in a variety of tasks. Niazi et al. outlined how digital pathology combined with AI can support quantitative biomarker assessment, triage, and quality assurance, positioning AI not as a replacement for the pathologist but as an adjunct layer that reduces variability and supports reproducible workflows. This paradigm has since been extended to multiple biomarkers, tumor types, and use cases ranging from routine quantification to advanced prognostic modeling [11,12].

Within this framework, several studies have specifically addressed automated or AI‑assisted scoring of breast cancer biomarkers. Shafi et al. integrated and validated automated digital image analysis for ER immunohistochemistry in a fully digital clinical workflow, demonstrating that algorithmic quantification can achieve high concordance with pathologist scoring while fitting within existing laboratory processes [13]. Abele et al. further showed noninferiority of AI‑assisted analysis for Ki‑67 and ER/PR in routine breast cancer diagnostics, suggesting that automated tools can safely support, and in some cases streamline, biomarker evaluation in daily practice [14]. More recently, Jung et al. reported that an AI analyzer augmented the interpretation of HER2, ER, and PR by improving interobserver agreement in a reader study of 201 cases, reinforcing the concept that algorithmic support is particularly beneficial in borderline and challenging cases. Collectively, these studies indicate that AI-based systems can enhance reproducibility and consistency of hormone receptor and HER2 scoring in real-world settings [15].

Beyond classical digital image analysis pipelines, AI has also been used to reduce interobserver variation in other IHC and morphologic tasks. For example, Choi et al. showed that an AI‑powered PD‑L1 analyzer reduced interobserver variation in the tumor proportion score for non‑small cell lung cancer while improving the prediction of immunotherapy response, illustrating how standardized quantitative outputs can enhance both diagnostic harmony and clinical utility [16]. In a separate study, deep learning was used to enhance tumor‑infiltrating lymphocyte evaluation and prediction of therapeutic response in breast cancer, again demonstrating that mathematically constrained, algorithmic evaluation can outperform or stabilize human estimation under complex visual conditions. At the diagnostic level, Raciti et al. validated AI‑augmented pathology for prostate cancer detection. They demonstrated significant gains in diagnostic accuracy, providing additional evidence that AI systems, when integrated into routine workflows, can elevate overall diagnostic performance rather than merely replicate human variability [17]. These experiences across biomarkers and tumor types collectively support the notion that AI can serve as a reproducible and generalizable ancillary tool for pathology practice.

The present study extends this body of work into a different, yet complementary, dimension: instead of introducing another task‑specific algorithm, we propose a reproducible protocol for deploying multimodal LLMs as ancillary tools for ER/PR scoring. The focus is deliberately methodological; our primary objective is to define how LLMs should be used through structured RPE and ZSR protocol, rather than to perform a traditional device‑like validation of a fixed model. By iteratively refining qualitative and semi‑qualitative prompts based on error analysis against CAP Proficiency Testing consensus cases, we demonstrate that prompt logic, intensity calibration, and explicit decision trees can systematically transform general‑purpose LLMs into constrained scoring engines for DAB‑based IHC. Importantly, once the prompts reached a stable configuration, all three evaluated models (Claude, Gemini, and a locally hosted Gemma) achieved high concordance with the CAP consensus despite architectural differences and parameter scale, emphasizing that the governing logical framework is more critical than raw model capacity.

An additional contribution of this work is the explicit treatment of conversational context as a controllable variable in LLM‑based image interpretation. During iterative development, we observed that processing multiple cases within a single session led to conversational inertia, with the model drifting toward self‑consistent patterns at the expense of objective accuracy, particularly around the 1-10% low‑positive threshold. The ZSR protocol, which mandates a fresh session for each evaluation round, effectively decouples individual cases and restores scoring independence, raising concordance from an initial 70% to expert‑level performance. This observation aligns with broader concerns in the LLM literature regarding context‑induced bias and hallucination. It suggests that explicit session‑reset rules may be as important as the prompt content itself when deploying LLMs in regulated diagnostic environments. Furthermore, by showing that a lightweight, locally hosted model can approximate the performance of cloud‑based systems once embedded within a robust RPE-ZSR framework, we provide a viable pathway for resource‑constrained laboratories to obtain AI support while addressing privacy and data‑governance constraints. From a practical standpoint, the protocol we describe is designed to be directly implementable using standard CAP proficiency tissue microarrays, widely available microscope-camera configurations, and current LLM interfaces.

**Figure 1:**
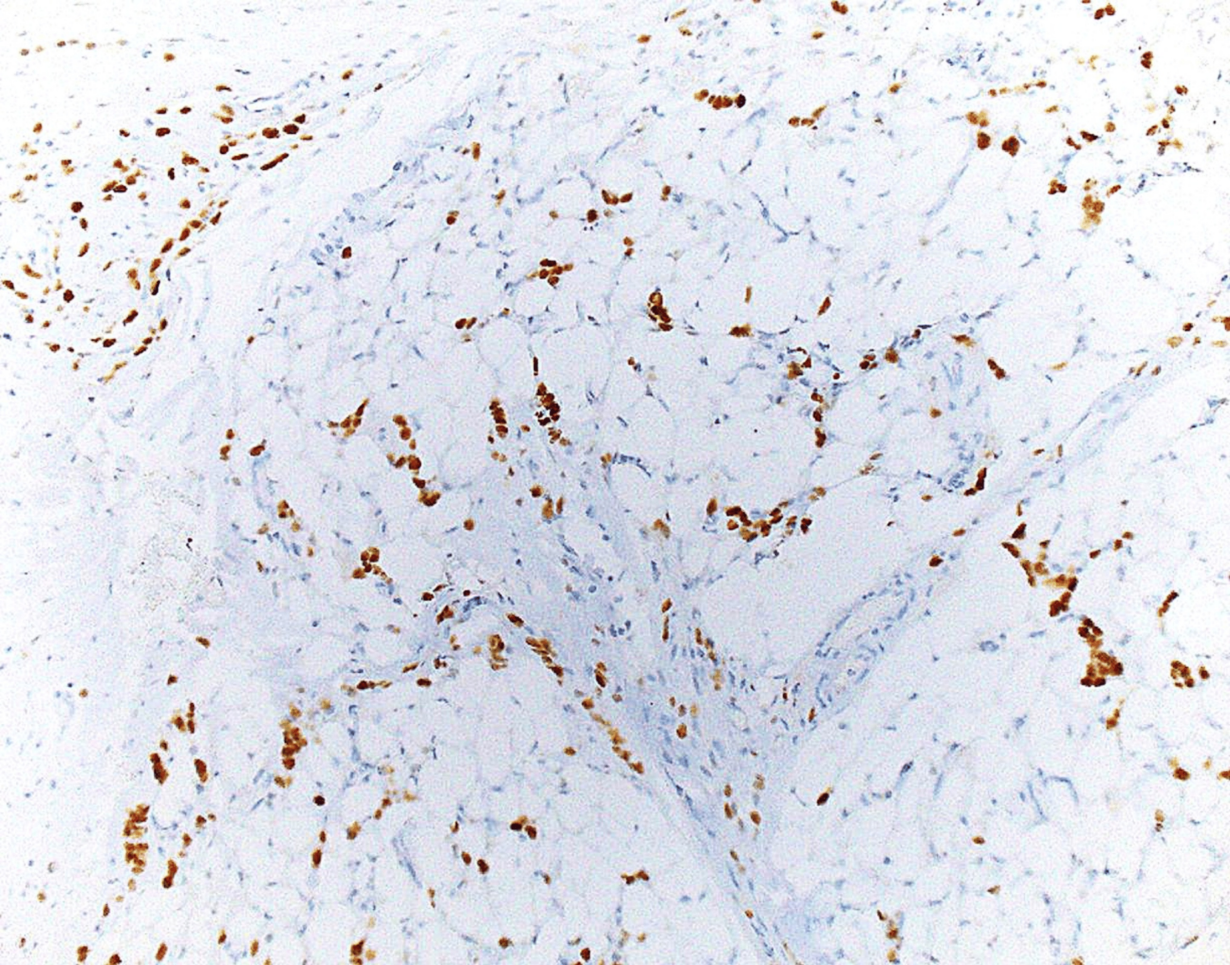
ER immunostain interpreted as 3+ (high) by CAP consensus but low or negative by LLMs, likely due to sparse cellularity being interpreted by LLMs as less than 1% ER: estrogen receptor, CAP: College of American Pathologists, LLMs: large language models

A key finding was the identification of specific technical artifacts that initially led the LLM to misclassify borderline images. In the critical 0% to 1% threshold, the primary drivers of error were image quality and pre-analytical slide preparation, low image resolution/compression, and improper brightness settings (over- or underexposure). Furthermore, the absence of proper white-balance adjustment during image acquisition often skewed the color temperature, leading the models to misinterpret background hues as legitimate staining. Another important factor was sparse cellularity in cases with a consensus score of 3+ based on staining intensity. Still, the LLM classified it as 1+ or even negative, believing that positive cells are in fact less than 1% (Figure 1). The prompt refinement protocol described in this article mitigated this issue by round 3 for the commercial LLM; however, it persisted for the locally hosted LLM Gemma.

The proposed methodology is intended not as a competitor to existing proprietary image analysis platforms but as a transparent, adaptable layer that can run on top of existing digital pathology infrastructure. By aligning the output categories with clinically meaningful tiers (e.g., negative, low expression, high expression) and using CAP consensus as the reference, the framework is readily integrated into current reporting structures and quality-assurance workflows.

This work should be interpreted primarily as a protocol- and framework-development study rather than a definitive clinical validation of LLM‑based ER/PR scoring. The relatively small dataset of 80 TMA cores, while providing a highly curated CAP consensus reference, limits generalizability and does not capture the full spectrum of staining heterogeneity, tumor morphology, and pre-analytical variability encountered in routine surgical pathology practice. This sample size is appropriate for a feasibility and protocol-development study but is insufficient for formal clinical validation. Image acquisition was limited to the JPEG format at a single magnification due to current LLM platform constraints, introducing compression‑related artifacts that may affect perceived chromogen intensity. The LLM prompts were optimized for three specific models and may require recalibration for other LLM architectures or future model versions. The ZSR protocol, while effective in mitigating conversational bias, adds workflow complexity that has not yet been optimized for high-throughput clinical deployment. A further limitation inherent to web-based LLM testing is platform impermanence: model weights, interface behavior, and image handling parameters may change over time without notice, reducing the long-term reproducibility of results obtained through consumer-facing interfaces. This reinforces the value of the prompt framework and protocol described here, which can be reapplied as model versions evolve. Consequently, independent replication across diverse institutions, staining protocols, and hardware configurations, ideally within formal validation studies, will be essential before this methodology can be adopted as a routine clinical decision support tool.

## Conclusions

This study demonstrates that LLMs can serve as reliable, reproducible ancillary tools for ER/PR IHC scoring in breast cancer pathology, provided they are deployed through a rigorously standardized framework. The key insight is that diagnostic accuracy is driven not by model size or architecture, but by systematic RPE and the ZSR protocol, the latter proving essential for eliminating conversational bias and elevating concordance with CAP consensus from a baseline of 70-80% to over 95%. Performance varied across models and scoring approaches, with Claude achieving the highest concordance (90-98%), followed by Gemini (85-100%) and Gemma (73-93%), reflecting differences in multimodal reasoning capacity that should be considered when selecting a model for implementation. Locally hosted models substantially mitigate HIPAA-related data governance risks and reduce infrastructure costs by processing images without transmitting patient data to external servers. However, formal HIPAA compliance determination remains institution-specific and outside the scope of this study.

Attention to pre-analytical variables, including slide cleanliness, image compression, and white balance, remains essential to minimize artifact-driven misclassifications. As digital pathology continues to mature, this protocol offers an immediately actionable, infrastructure-light pathway toward a more standardized and globally equitable diagnostic framework for hormone receptor assessment in breast cancer patients, pending multi-institutional validation.
